# Iron in the Tumor Microenvironment—Connecting the Dots

**DOI:** 10.3389/fonc.2018.00549

**Published:** 2018-11-26

**Authors:** Christa Pfeifhofer-Obermair, Piotr Tymoszuk, Verena Petzer, Günter Weiss, Manfred Nairz

**Affiliations:** ^1^Department of Internal Medicine II, Infectious Diseases, Immunology, Rheumatology, Pneumology, Medical University of Innsbruck, Innsbruck, Austria; ^2^Christian Doppler Laboratory for Iron Metabolism and Anemia Research, Medical University of Innsbruck, Innsbruck, Austria

**Keywords:** iron, anemia of cancer, ACD, hepcidin, ferroptosis, TAM

## Abstract

Iron metabolism and tumor biology are intimately linked. Iron facilitates the production of oxygen radicals, which may either result in iron-induced cell death, ferroptosis, or contribute to mutagenicity and malignant transformation. Once transformed, malignant cells require high amounts of iron for proliferation. In addition, iron has multiple regulatory effects on the immune system, thus affecting tumor surveillance by immune cells. For these reasons, inconsiderate iron supplementation in cancer patients has the potential of worsening disease course and outcome. On the other hand, chronic immune activation in the setting of malignancy alters systemic iron homeostasis and directs iron fluxes into myeloid cells. While this response aims at withdrawing iron from tumor cells, it may impair the effector functions of tumor-associated macrophages and will result in iron-restricted erythropoiesis and the development of anemia, subsequently. This review summarizes our current knowledge of the interconnections of iron homeostasis with cancer biology, discusses current clinical controversies in the treatment of anemia of cancer and focuses on the potential roles of iron in the solid tumor microenvironment, also speculating on yet unknown molecular mechanisms.

## Introduction

There are numerous interconnections between iron homeostasis and cancer biology. However, our knowledge of many of these links is largely descriptive and based on data obtained from *in vitro* models using immortalized cell lines or from *in vivo* animal models employing xenogeneic tumor cell transplantation. Many of the potential roles of iron in cancer, generally, and in the tumor microenvironment (TME), specifically, have therefore not been formally addressed in human tumor entities and patient cohorts yet.

One aspect of the interconnection between iron and cancer is based on the fact that excess labile iron is toxic and catalyzes the formation of reactive oxygen species (ROS) via Fenton-/Haber-Weiss chemistry (1). As a consequence, iron may drive the malignant transformation of cells by directly damaging DNA, eventually leading to mutagenic transformation, or through protein and lipid modifications within malignant cells, resulting in more aggressive tumor behavior (2). When iron-dependent lipid peroxidation exceeds the cell's glutathione-mediated anti-oxidative defense capacity, inactivation of glutathione peroxidase (GPX)-4 culminates in a unique form of iron-induced cell death known as ferroptosis (3). On the other hand, proliferation of neoplastic cells regularly occurs at an enhanced rate, requiring increased iron supply because DNA replication is an iron-dependent process (4, 5). DNA polymerases and helicases contain iron-sulfur groups, rendering DNA replication one of the numerous synthetic and metabolic pathways that rely on iron as essential co-factor (6). Therefore, the availability of iron to tumor cells may affect either cell survival or growth rate and the course of disease, consequently. In addition, cellular iron availability impacts on mitochondrial respiration, ATP (for adenosine triphosphate) and mitochondrial radical formation, but also controls cellular metabolism and aerobic glycolysis via its regulatory effects on citric acid cycle enzymes (7, 8). In addition, neovascularization is affected by iron because of its impact on hypoxia inducible factor (HIF) activation and vascular endothelial growth factor (VEGF) production and on the function of endothelial cells (EC) (9, 10). Also, tumor-associated macrophages (TAMs) and EC diversely interact in the TME, and some of these interactions are modulated by iron availability, impacting on tumor progression and metastasis formation (11–16).

Cancer biology and immune surveillance are inseparably interconnected (17). A central nexus of this linkage is the competition for iron between neoplastic cells and the immune system which takes place both at the systemic level and in the microenvironment (18). Presumably, immune-driven adaptations of iron homeostasis in the presence of inflammatory stimuli have evolved during evolution as mechanisms to fight off bacteria and other pathogens, most of which require iron as essential growth factor (19–21). However, similar regulations occur when cancer cells are detected by the immune system because pathogen-associated molecular patterns (PAMP) and danger-associated molecular patterns (DAMP) elicit identical responses. The adaptation of systemic iron homeostasis to these inflammatory stimuli is orchestrated by soluble mediators including cytokines, such as interleukin (IL)-6 and acute-phase reactants, such as hepcidin and α1-antitrypsin (22–27). In addition, ROS and reactive nitrogen species (RNS), generated to damage cancer cells, also affect the way immune cells handle iron at the systemic level and in the TME (28, 29). Increased iron uptake into myeloid cells along with reduced iron export result in iron storage and sequestration in the mononuclear phagocyte system (MPS). Iron accumulation in the MPS may affect innate immunity in either direction. Typically, T helper type-1 (T_H_1)-driven pathways are inhibited by macrophage iron overload (IO), whereas ROS-induced pro-inflammatory signaling events are stimulated by iron (30). Which of these pathways predominate in anti-tumor immunity remains to be determined, though, because many results have been obtained in non-neoplastic inflammatory models (31–34). As a side effect or iron sequestration in the MPS, this trace element is less available for hemoglobin (Hb) synthesis by erythroid progenitors (EPs) in the bone marrow. Taken together, multiple mechanisms contribute to the alterations of iron homeostasis observed in cancer patients, which progress to clinically evident anemia of cancer (AOC).

AOC is extremely common and occurs in ~40–70% of cancer patients (35, 36). Importantly, the anemia affects organ function, and a higher degree of AOC is associated with reduced quality of life and survival of cancer patients (37, 38). Therefore, treatment of AOC is warranted but the benefit-to-risk ratio has to be carefully considered on an individual basis because therapy-associated effects on the underlying malignancy have been observed, too. For example, treatment with iron, erythropoiesis-stimulating agents (ESAs) or packed red blood cells (RBCs), administered to treat or correct the AOC, all carry the potential to promote tumor cell proliferation or impair anti-tumor immunity and have been associated with a shortened survival or recurrence of cancer (39–41). Theoretically, this is also true of novel therapeutic options for anemia of chronic disease (ACD) or AOC, especially the ones that target the hepcidin-ferroportin (FPN)-1 axis or HIF activation. On the other hand, many cancer cells rely on GPX4 to evade ferroptosis, rendering it an attractive target for tumor therapy. To avoid unintended effects, we need prospective clinical outcome data from rigorously conducted prospective randomized controlled trials (RCTs) as well as a more profound understanding of the multiple interconnections between iron metabolism and tumor occurrence and progression. This review provides an overview of our current knowledge of some of these interconnections.

## Ferroportin-1 forms the “iron gate” to the circulation

**Figure 1:**
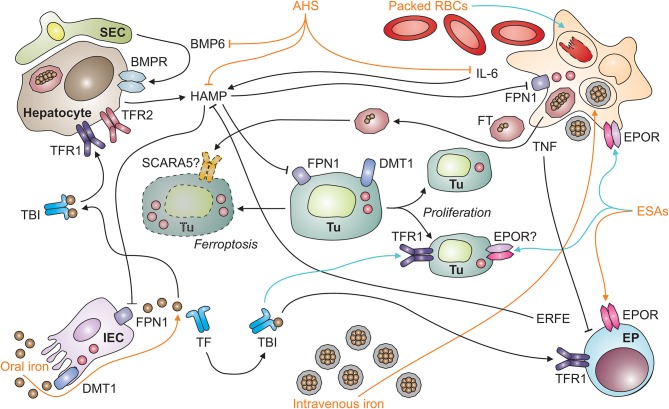
Systemic iron homeostasis in malignancy and potential effects of therapeutic intervention. After absorption by intestinal epithelial cells (IECs; depicted in the left lower corner) in the duodenum and upper jejunum, iron is loaded onto transferrin (TF) and distributed throughout the body as TBI (for transferrin-bound iron). TBI levels in the circulation are sensed by hepatocytes (depicted in the left upper corner) via transferrin receptors-1 and−2 (TFR1 and TFR2). An increase in iron levels in plasma results in the secretion of hepcidin antimicrobial peptide (HAMP). HAMP is also induced upon tissue iron loading, which results in the release of bone-morphogenic protein (BMP)-6 by liver sinusoidal endothelial cells (SECs; left upper corner). In addition, the cytokine interleukin-6 (IL-6) stimulates HAMP production by hepatocytes in inflammatory conditions, such as neoplasia. HAMP binds to ferroportin (FPN)-1 and blocks its iron export function, particularly in macrophages (MΦ; right upper corner), which results in iron sequestration and storage in ferritin (FT). FT can also be secreted by macrophages and taken up from plasma via specific receptors, such as SCARA5 (for scavenger receptor class A member-5). In the setting of malignancy, macrophages and other types of immune cells also secrete tumor necrosis factor (TNF). Among numerous functions, TNF inhibits the proliferation of erythroid progenitors (EP; right lower corner) in the bone marrow and their responsiveness to erythropoietin (EPO) produced in the kidneys (not depicted). Therapeutic options (depicted in orange) for cancer-related anemia include oral and intravenous iron preparations, anti-hepcidin strategies (AHS), erythropoiesis-stimulating agents (ESAs), and packed red blood cells (RBCs). All of these medications have potential side effects (depicted in turquois) on immune cells. For example, intravenous iron and packed RBCs can result in macrophage iron overload and impair their anti-tumor immune functions or facilitate the proliferation of tumor cells (Tu; depicted in the center, including dying [left-hand side] and proliferating [right-hand side] tumor cells). Cell types are indicated in bold; processes in italic. BMPR, BMP receptor; DMT1, divalent metal transporter-1; ERFE, erythroferrone; EPOR, EPO receptor.

Iron homeostasis is tightly maintained because too little iron impairs cell metabolism and function whereas too much iron is potentially toxic (42, 43). In mammals, the major regulated step in systemic iron homeostasis is its transfer to the circulation at sites of iron absorption or recycling because no controlled excretory mechanism exists (44). The principal protein to mediate iron transfer from cells to the circulation is FPN1, the only ferrous iron exporter known. FPN1 is highly expressed in iron-recycling macrophage populations, such as red-pulp macrophages (RPMs) in the spleen and Kupffer cells (KCs) in the liver, at the basolateral surface of absorptive intestinal epithelial cells (IECs) in the duodenum and proximal jejunum and in the synzytiotrophoblast (45, 46). FPN1 mediates efflux of ferrous iron and cooperates with either of three multi-copper oxidases able to convert ferrous iron to its ferric form, i.e., hephaestin, ceruloplasmin, and zyklopen (47, 48). In the extracellular space, ferric iron is bound by apo-transferrin (TF). TF is the key iron transport protein in plasma, accepts one or two iron atoms per molecule and distributes them as holo-TF throughout the body for cellular uptake by transferrin receptor (TFR)-1 and utilization, for example by the erythron (Figure 1).

Given its position as a gatekeeper to the circulation, FPN1 expression is controlled by several mechanisms ranging from transcriptional to post-translational regulation: (i) Iron-containing heme moieties stimulate FPN1 transcription via the stress-sensitive transcription factor Nrf2 (for nuclear factor (erythroid-derived 2)-like 2) (49), while increased iron levels in cells can also mitigate FPN1 transcription by a thus far not specified mechanism (50, 51). (ii) FPN1 mRNA translation is fine-tuned by two miRNAs, miR-485-3p and miR-20a (42, 52). The latter mechanism may be of special relevance for tumor cells as exemplified by the fact that in non-small cell lung cancer (NSCLC), increased miR-20a levels are found in biopsies, and miR-20a represses FPN1 expression and enhances iron availability for *in vitro* proliferation (53). (iii) An iron-responsive element (IRE) is present in the 5′ untranslated region of FPN1's mRNA. Iron deficiency (ID) in the cytoplasm is detected by iron-regulatory proteins (IRPs)-1 and−2, which enhances their activity to bind to the IRE and thereby inhibit FPN1 translation. By virtue of this mechanism, iron export is turned off when intracellular iron is already scarce. The IRP/IRE system appears to be relevant for tumor cell proliferation, too, in that activation of IRP2 along with an increase in iron content is documented for prostate cancer cells *in vitro* (54). Unexpectedly, overexpression of IRP1 impairs the growth of lung cancer cells transplanted into nude mice despite increased TFR1 levels (55). (iv) Most importantly, the transport activity of FPN1 is regulated post-translationally by its ligand hepcidin (Figure 1).

## Hepcidin levels regulate systemic iron homeostasis

Hepcidin is the body's iron-regulatory hormone and it acts in a negative feedback manner: Hepcidin binds to FPN1, closes its iron transport pore and induces its retraction from the cell surface with the subsequent induction of its degradation, thus blocking cellular iron export via FPN1 (45, 56). Therefore, high hepcidin levels, as observed in malignancy-driven inflammation, reduce iron transport to the circulation and cause iron retention in the MPS. Hepcidin is primarily formed and secreted by hepatocytes and its expression underlies multiple regulatory mechanisms which integrate the partly opposing measured variables that indicate both, systemic iron supply and demand. For example, an increase in body iron stores stimulates bone morphogenic protein (BMP)-6 production by sinusoidal EC in the liver (57) (Figure 1). BMP6 then acts on adjacent hepatocytes and induces hepcidin via the SMAD (homologs of Sma and Mad (mothers against decapentaplegic) proteins) pathway (58). In contrast, elevated serum iron levels act on hepatocytes themselves which carry a sensory protein complex of TFR1, TFR2, HFE and hemojuvelin (HJV) on their cell surface (59). Iron sensing by this complex also activates SMAD signaling, and hepcidin transcription, consequently (60, 61). Therefore, increasing serum iron levels are rapidly sensed by hepatocytes and balanced out by hepcidin secretion. Proinflammatory cytokines typically stimulate hepcidin expression, too. This effect is best described for IL-6. Specifically, IL-6, produced by macrophages and many other cell types, induces STAT3 (for signal transducer and activator of transcription-3) phosphorylation and thus hepcidin transcription, which contributes to the pathogenesis of ACD, as outlined below (62–64).

In contrast to inflammatory signals, absolute ID attributable to bleeding episodes or insufficient iron absorption, results in a decrease of hepcidin levels. This downregulation promotes iron absorption and re-distribution from the MPS to secure iron delivery for Hb synthesis and cellular functionality (65–67). In the setting of anemia, additional factors contribute to a reduction of hepcidin synthesis. A central repressor is erythroferrone (ERFE), a mainly erythropoietin (EPO)-inducible protein secreted by EPs (68–70). Hepcidin also needs to be repressed in hypoxia so that iron can be directed to erythropoietic progenitors for Hb synthesis (71). Central factors suppressing hepcidin production in anemia or hypoxic conditions include EPO and growth differentiation factor (GDF)-15 as well as platelet-derived growth factor (PDGF)-BB, the latter inhibiting hepcidin transcription via CREB-H (for cyclic AMP response element-binding protein H) (71–75). The transcription factor CREB-H is also essential to induce hepcidin in response to endoplasmatic reticulum (ER) stress, and this transcriptional induction is modified by CHOP (for CCAAT-enhancer-binding protein homologous protein), STAT3 and SMAD5 (76, 77, 78). Because ER stress is also exerted by chemotherapeutics, ER stress-sensitive transcription factors provide another potential link between iron metabolism and cancer (79, 80). In conclusion, FPN1 and hepcidin form a functional unit that constitutes the central regulator of systemic iron homeostasis both under physiological and pathological conditions including cancer.

## Systemic iron homeostasis in tumor patients

The recognition of tumor cells and of neoantigens provides a strong stimulus for the immune system (81, 82). Both, innate immune mechanisms and T cell responses against malignant cells result in the production of a myriad of cytokines, such as interferon (IFN)-γ, tumor necrosis factor (TNF), IL-1ß, IL-6, IL-10, IL-13, and IL-22, all of which also impact on iron fluxes in the MPS contributing to the development of hypoferremia and hyperferritinemia as typical immune-driven alterations of iron metabolism (83–90). For example, IFN-γ, produced by T_H_1 cells, NKT (for natural killer T) cells and other cell types, increases DMT1 (for divalent metal transporter-1) expression in the MPS while decreasing FPN1 levels, thus contributing to iron sequestration (91). TNF, secreted by many cell types including T_H_1 cells, monocytes and macrophages, has similar effects, causing an imbalance between iron import and export in favor of the former, whereas IL-10 may mainly stimulate ferritin (FT) translation and thus iron storage (84, 91, 92). These alterations divert iron fluxes into the MPS and make it unavailable for erythropoiesis, a condition known as functional ID (93), a major hallmark of AOC.

## Anemia of cancer

Both IFN-γ and TNF have other pleiotropic effects and also inhibit EPO expression in the kidney and erythropoiesis in the bone marrow, aggravating the degree of AOC (94, 95). The latter effects are based on the fact that IFN-γ inhibits the proliferation of EPs and that TNF induces ROS, which damage them (96). Moreover, it is feasible to assume that these and other mediators also cause functional alterations in the bone marrow microenvironment, for instance by shifting hematopoiesis toward the myelopoietic direction or by impairing iron transfer from erythroid island macrophages to EPs. In addition, absolute ID may be present in cancer patients because of bleeding episodes, for example from malignant ulcers in the gastrointestinal or urogenital tract, extensive blood sampling, surgery or other interventions (97, 98). Furthermore, many other mechanisms may contribute to the development of AOC depending on the specific tumor entity. In multiple myeloma (MM), for example, IL-6 is produced by stromal cells and malignant cells themselves within the bone marrow and it appears to stimulate hepcidin production distantly in the liver (99). Another pathogenetic factor often contributing to the occurrence of AOC is the infiltration of the bone marrow by malignant cells which may affect erythropoiesis by nutrient and space deprivation, by damage to hematopoietic stem cells and by disruption of the integrity and function of erythroid islands and stem cell niches (40). These mechanisms may be most relevant in hematopoietic malignancies, such as acute leukemia in which a rapid expansion of malignant clones in the bone marrow takes place. Also, radiotherapy and chemotherapeutics have toxic effects on the bone marrow, thereby affecting all lines of hematopoiesis including RBC production and circulatory half life. Moreover, disease- or therapy-associated hemolysis or microangiopathy, malnutrition and comorbidities, such as chronic kidney disease (CKD) can contribute to AOC and affect its frequency and severity.

In summary, the AOC can be considered a subgroup of the ACD because of their similar pathophysiology. AOC is extremely common, occurs in various types of malignancy and is present in ~40–70% of cancer patients (35, 36). Its prevalence is affected by the type of cancer and by anti-tumor treatment and, if left untreated, it worsens as the disease progresses. Importantly, the AOC is associated with a shorter survival of cancer patients, as its occurrence and severity is associated with a more advanced disease (37, 38).

## Treatments options for cancer-related anemia

Anemia may negatively impact on the quality of life and cardio-vascular performance of cancer patients, necessitating treatment of AOC. To date, we lack information on whether correction of the anemia exerts beneficial, neutral or detrimental effects toward the course of the underlying malignant disease. Of note, the pivotal therapy of AOC is the cure of the causative malignancy, which often results in resolution of anemia over time. As this may not always be feasible, other treatments have been used or are in development to correct Hb levels. Therapeutic options for the AOC include ESAs alone or combined with iron supplementation, blood transfusions and newly emerging treatment options with anti-hepcidin strategies (AHS) and PHD (for prolyl HIF dioxygenases) inhibitors (Figure 1).

In the setting of cancer and normal kidney function, ESAs help to overcome the resistance of the erythron to EPO. This growth factor is often suppressed by cytokines and, in the setting of AOC, circulates at concentrations too low for the degree of anemia. However, EPs are not the only cell types responsive to EPO. EPO and ESAs also may have extra-erythropoietic effects because the EPO receptor (EPOR) is expressed by many cell types other than EPs (100). EPOR has also been detected on many malignant cells, including NSCLC and breast cancer cells, although some controversy exists on the specificity of antibodies used to detect EPOR by means of immunohistochemistry (101–104). In breast cancer, there appears to be a negative correlation between EPOR expression and disease outcome, possibly because EPO counteracts p53-dependent apoptosis through induction of anti-apoptotic Bcl-X_L_ (for B-cell lymphoma-extra large) (105, 106). In addition, EPO may stimulate the expression of TFR1, which increases TBI uptake into cells and can enhance the availability of iron for proliferation of malignant cells (107). EPOR is also present on immune cells including macrophages, B cells and T cells and may therefore exert diverse immune-modulatory effects (108, 109). In macrophages, EPOR ligation inhibits the activation of the NF-κB (for Nuclear factor kappa-light-chain-enhancer of activated B cells) subunit p65. As a consequence, pro-inflammatory effector pathways are impaired in EPO-stimulated macrophages (110). Similarly, EPO also reduces the autoreactivity of T cells. On the other hand, EPO beneficially affects the B and T cell responses against malignant cells in a mouse model of MM, suggesting differential effects of EPO and EPOR in various disease entities (111, 112).

PHD inhibitors form a novel class of drugs for the treatment of anemia. Their efficacy has been investigated in patients with CKD. However, their safety profile in cancer patients or in elderly individuals at increased risk of cancer remains to be assessed. A recent work dealing with the safety and efficacy of the PHD inhibitor roxadustat in a murine model of spontaneous mammary carcinoma demonstrates an improvement of cancer-related anemia together with increased VEGF production in the malignant tissue. In spite of that, no net boosting of tumor onset or progression is observed. The lack of reports concerning other tumor types and safety of iron and PHD application calls for further research and the long-term outcomes of currently ongoing phase III studies with different PHD inhibitors are awaited (66).

## Transfusion-related immune modulation

Blood transfusions may affect the outcome of cancer, too (113–115). This may be attributable to the fact that packed RBCs inevitably contain damaged cells and other immune-modulatory compounds. Stringent quality control, short-term storage and the depletion of leukocytes and platelets aim at reducing the risk of transfusion-related immune modulation (TRIM). Nevertheless, RBC damage may occur during and after transfusion, for instance due to mechanical stress or minor blood group incompatibilities, which releases free Hb, heme and iron. Free Hb impairs immunity and is a predictor of mortality in patients with sepsis and during extracorporal membrane oxygenation, partly because free heme elicits apoptosis (116–118). However, whether these observations are also relevant to other inflammatory conditions, such as neoplasia is unknown. Similarly, NTBI is implicated in TRIM in preterm infants, but this requires verification in adults and in the setting of malignancy (119). RBC transfusions may, however, directly deliver various forms of iron to tumor cells. Furthermore, RBCs themselves affect immune cell functions. For example, they inhibit T cell proliferation via direct cell-cell contact and affect the functions of dendritic cells (DCs) (120, 121). Importantly, monocytes and iron-recycling macrophages in the spleen and liver take up damaged RBCs via scavenger receptors, degrade Hb using lysosomal enzymes and heme oxygenase (HMOX)-1 and export iron via FPN1 (122, 123). FPN1^+^ iron-recycling monocytes may provide iron to tumor cells in their vicinity, especially in the liver, where these monocytes reside in large quantities, or after loading of ferric iron onto TF. In addition to RBCs and their compounds, anti-inflammatory cytokines and immune-modulatory extracellular vesicles may be present in blood products and affect anti-tumor immunity either systemically or in the TME. Therefore, both soluble and cellular factors may contribute to the immune-modulatory effects of RBC transfusions in cancer (Figure 1).

## Iron supplementation in cancer patients

Iron supplementation is a treatment option for anemic cancer patients with absolute ID. In the setting of immune activation secondary to cancer or other diseases, though, absolute ID is difficult to define as higher cut-offs for serum FT have to be employed. To date, no gold standard biomarker test is available which can differentiate between absolute and functional ID in the setting of inflammation (124–126). However, current clinical practice guidelines propose that a serum FT < 100 ng/ml indicates absolute ID and a TSAT < 20% together with a serum FT > 100 ng/ml characterizes functional ID in cancer patients (127). Oral iron formulations often cause gastrointestinal side effects and may be ineffective in AOC, especially when hepcidin levels are high, limiting oral bioavailability (128, 129). In addition, *in vitro* experiments suggest that at least two forms of oral iron, ferric citrate and ferric EDTA (for ethylenediaminetetraacetic acid), at supra-physiological concentrations can activate the EGF (for epidermal growth factor) receptor and MAP (for mitogen-activated protein) kinase signaling cascade in colon cancer cells, suggesting that these seemingly harmless compounds may have oncogenic potential when used at excess dosages (130). Intravenous iron preparations are an alternative to oral iron and constitute iron-carbohydrate nanoparticles which are engulfed and handled by myeloid cells (131) (Figure 1). Efficacy and long-term safety of intravenous iron formulations for the treatment of AOC are still under debate and should be evaluated in prospective RCTs. Most studies available suggest that intravenous iron supplementation in solid tumor patients is well-tolerated, replenishes iron stores and increases Hb levels, thus reducing the need for ESA or blood transfusion (132). However, data on effects of iron supplementation on tumor biology and long-term outcome of cancer patients are still scarce and a matter of serious concern. Until such data become available, a restrictive iron supplementation regimen is warranted for symptomatic patients with AOC but application thresholds for Hb and FT remain to be defined based on clinical data (133, 134).

## Anti-hepcidin strategies (AHS)

AHS target hepcidin production, its circulatory concentrations or its effect on FPN1, thereby aiming to restore iron delivery from the MPS to the circulation. While these effects mean to provide iron for erythropoiesis, they also may supply iron to tumor cells or affect anti-tumor immunity. As for hepcidin production, BMPR/SMAD and IL-6R/STAT3 signaling constitute two attractive pathways to pharmacologically suppress hepcidin production.

*In vitro*, BMP6 has both stimulatory and inhibitory effects on tumor cells. BMP6 facilitates bone metastasis in an *in vivo* model (135). By contrast, treatment with BMP6 inhibits proliferation of breast cancer cells *in vitro* (136). Moreover, BMP6, secreted by prostate cancer cells, stimulates macrophages to produce IL-1α which in turn promotes tube formation by EC, a prerequisite for tumor neovascularization (137). Furthermore, BMP6 stimulates RNS production by macrophages but this has not been reported for TAMs yet. Given BMP6's opposing effects, neutralizing antibodies administered to treat AOC may affect the underlying malignancy in either direction. Similar to BMP6 neutralization, BMPR phosphorylation can be inhibited to decrease hepcidin production. The small molecule LDN-193189 is efficient in doing so and ameliorates ACD in a non-neoplastic rat model (27). ERFE analogs or other hypoxia- or erythropoiesis-driven inhibitors of hepcidin production, such as PDGF-BB, GDF-15 or PHD inhibitors may have similar effects and may become promising treatment options for ACD and AOC. Notably, endogenous heparins inhibit BMP/SMAD signaling, too. Based on this observation, heparin derivatives that lack anti-thrombotic but retain hepcidin-repressing effects have been developed. These compounds are effective in the treatment of ACD (138). Again, no prospective clinical data are available on the course of the underlying malignancy when hepcidin is suppressed to treat AOC.

Blockade of IL-6 signaling with tocilizumab, a humanized antibody directed against its cytokine receptor, is efficient in treating ACD in an animal model of arthritis and in Castelman's disease patients in an RCT (139, 140). In a retrospective analysis of patients with rheumatoid arthritis, long-term treatment with tocilizumab did not affect the expected rate of malignancies (141). Furthermore, the concerted blockade of IL-6 receptor and IL-8 receptor inhibits breast cancer metastasis in mouse xenograft models, suggesting that inhibition of hepcidin induction via the IL-6/STAT3 pathway may be a rather safe approach to treat AOC, at least in some tumor entities (142).

Direct neutralization of hepcidin can be achieved with antibodies, anticalins, or Spiegelmers, all of which are being evaluated for the treatment of ACD. Thus far, the hepcidin-neutralizing antibody LY2787106 is efficient in mobilizing iron in the setting of AOC (143). However, without further follow-up, the long-term safety of either of these treatment strategies for cancer patients is difficult to predict. Therefore, we need large RCTs with relevant clinical endpoints to assess the efficacy and safety of recently developed treatment options for AOC and to define optimal therapeutic start- and endpoints in a prospective fashion.

## A quadriga of iron-binding proteins is present in plasma

**Figure 2:**
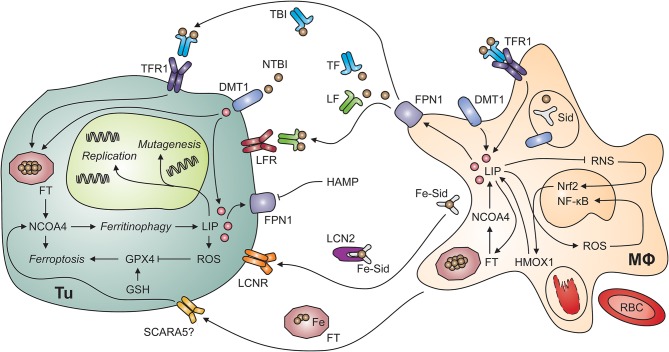
Putative interactions of tumor cells and tumor-associated macrophages. Myeloid cells, including tumor-associated macrophages (MΦ; right-hand side) possess multiple mechanisms to acquire iron including the uptake of non-transferrin-bound iron (NTBI) via divalent metal transporter (DMT)-1, endocytosis of transferrin-bound iron (TBI) via transferrin receptor (TFR)-1 and phagocytosis of aged or damaged red blood cells (RBCs). RBCs contain large amounts of iron incorporated in hemoglobin. The degradation of hemoglobin by proteases and heme oxygenase (HMOX)-1 releases free iron into the labile iron pool (LIP). An increase in the LIP then facilitates the production of reactive oxygen species (ROS), which activate nuclear factor-kappa B. In contrast, labile iron impairs the generation of reactive nitrogen species (RNS) via nitric oxide synthase-2. In the extracellular space, iron is present in at least four molecular forms, i.e., bound to transferrin (TF), bound to lactoferrin (LF), bound to siderophores (Sid) and lipocalin (Lcn)-2, and incorporated in ferritin (FT). All these forms may supply iron to tumor cells (Tu; left-hand side) because of their expression of specific receptors, possibly including the scavenger receptor A member (SCARA)-5, which binds FT. In addition, non-transferrin-bound iron (NTBI) may be present in the tumor microenvironment and acquired via DMT1. Once in the tumor cell's cytosol, labile iron can stimulate cell growth and DNA replication, induce mutagenesis or result in ferroptosis. Ferroptosis is a specific form of programmed cell death which is initiated by an increase in the LIP and in ROS production. ROS inactivate glutathione peroxidase (GPX)-4 after depletion of glutathione (GSH). Similarly, the degradation of FT by ferritinophagy, an autophagic process requiring the FT chaperone NCOA4 (for nuclear receptor coactivator-4), frees iron and can induce ferroptosis. On the other hand, iron can also be exported from the cytosol via ferroportin (FPN)-1 on tumor cells. However, this process is reduced when levels of hepcidin antimicrobial peptide (HAMP) in the circulation or in the tumor microenvironment are high. Cell types are indicated in bold; processes in italic. DNA, deoxyribonucleic acid; Nrf2, nuclear factor (erythroid-derived 2)-like-2.

There are at least four iron-binding proteins in plasma which carry the potential to either deliver iron to tumor cells or deprive it from them: TF, lactoferrin (LF), FT, and lipocalin (LCN)-2 can bind iron in different forms and their content of and affinity for iron as well as the distribution of corresponding cell surface receptors between normal nucleated cells and tumor cells may determine whether the latter can benefit from these proteins as iron sources (Figure 2). In addition, non-protein bound labile iron, not detectable in healthy individuals, can occur in the extracellular space in malignancies, especially in patients with acute myeloid leukemia or myelodysplastic syndrome (MDS) during conditioning for allogenic hemopoietic stem cell transplantation (144, 145).

Since TF is the key iron transport protein circulating in plasma, TFR1-mediated iron uptake is a simple and efficient iron acquisition strategy for malignant cells (Figure 2). Not surprisingly, overexpression of TFR1 has been found in many tumor entities including breast cancer, esophageal cancer, melanoma and glioblastoma cells (146–149). In addition, compounds targeting TFR1 are under consideration for molecular imaging and therapy of various tumor entities including B and T cell lymphomas (150, 151).

LF is a TF homolog with two binding site for ferric iron whose affinity exceed TF's by far (152). It is the predominant iron-binding protein in breast milk, saliva and tear fluid but also present in plasma. LF delivers iron to cells expressing specific receptors, one of which is GAPDH (for glyceraldehyde-3-phosphate dehydrogenase), a multifunctional protein present in the cell membrane of macrophages and other cell types (153, 154). GAPDH can also be secreted for subsequent autocrine uptake of LF or TF into cells (154, 155). Although malignant cells express GAPDH, uptake of LF-bound iron may be of minor importance for their iron supply because LF's predominant effect on breast, colorectal and other cancer cells *in vitro* is to impair signaling and cell cycle progression, to inhibit proliferation and to induce cell death (156–158). In line with these anti-neoplastic effects, mice lacking LF are more susceptible to dysplasia in the colon elicited by chronic inflammation (159). In addition, LF affects immune cell function and the composition of the intestinal microbiome but the impact of these observations on cancer biology are unknown (160, 161). However, it is interesting to note that a conjugate of LF and doxorubicin has beneficial effects in a prostate cancer model *in vivo* (162). Taken together, LF appears to have diverse anti-neoplastic effects and its therapeutic potential warrants further studies.

Macrophages secrete FT, which subsequently circulates in plasma in an iron-poor form (163). Serum FT can deliver iron to cells which carry corresponding receptors on their surface (Figure 2). TFR1 mediates binding and uptake of ferritin heavy chain (FTH) by some human cell types including erythroid cells, and TIM2 (for T cell immunoglobulin and mucin domain protein-2) fulfills a similar function in mouse cells (164, 165). Analogously, SCARA5 (for scavenger receptor class A member 5) is the receptor for FTL (166). Tumor cells may express FT receptors allowing them to utilize serum FT as iron source. Nevertheless, the expression of SCARA5 on tumor cells inhibits cell proliferation and migration because it impairs several signaling events including VEGF expression and ERK (for extracellular signal-regulated kinases) activation (167). High serum FT levels in cancer patients are associated with more aggressive clinical course and poor treatment response but it is unknown whether high FT actively contributes to disease progression or is a biomarker reflecting systemic immune activation (168), which is well-known to be associated with a poor prognosis of cancer (169).

LCN2 may contribute to both iron uptake and iron release by several cell types including cancer cells and TAMs (Figure 2). Among many functions, LCN2 acts as soluble scavenger for siderophores (170, 171). Siderophores are small iron-chelating molecules originally identified in bacteria but also produced by higher organisms including fungi and mammals (172–174). They bind ferric iron with extraordinarily high affinity and catecholate-type siderophores themselves are bound by LCN2. Thereafter, complexes of iron, a catecholate-siderophore and LCN2 are recognized by cell surface receptors including LCNR and megalin and thus mediate iron import into cells, which may contribute to the survival of thyroid carcinoma and other malignant cells (175, 176). However, LCNR has the intriguing property of providing bi-directional iron transport capacity because it can also accept intracellular LCN2 after binding of an iron-laden siderophore in the cytoplasm (177). Due to the fact that the oncogenic tyrosin kinase BCR-ABL (for breakpoint cluster region-Abelson) represses LCNR expression, iron-deprivation via the LCN2-LCNR pathway is inactive in BCR-ABL^+^ CML (chronic myeloid leukemia) cells, rendering them resistant to apoptosis (178). Expression of LCN2 is also associated with epithelial-mesenchymal transition (EMT), invasion, progression and metastatic spread of many types of cancer cells including those of breast, cholangiocellular, intestinal or prostate origin but model-specific differences exist (179–183). In addition, LCN2 forms a complex with matrix metalloproteinase (MMP)-9 (184). This stabilizes MMP9 activity and may contribute to metastatic spread (185). In mice with breast cancer, the oncogene HER (for human epidermal growth factor receptor) induces LCN2 expression and antibody-mediated blockade by trastuzumab antagonizes this effect (186). In prostate cancer cells, NF-κB as well as mutation or loss of p53 result in enhanced LCN2 expression (187). In addition, pro-inflammatory cytokines, such as TNF, IL-1ß, and IL-17 stimulate LCN2 expression via NF-κB (188, 189). The latter pathway opens the possibility that the immune response directed against tumor cells in fact induces a LCN2-dependent survival strategy but the biological or clinical significance of this putative paracrine mechanism has not been experimentally addressed yet. On the other hand, LCN2 has immune-modulatory effects itself which may help to limit tumor growth (190–195). Of note, LCN2 also stimulates VEGF expression via HIF and thus tumor angiogenesis (196). It is possible that LCN2-mediated iron depletion is an underlying mechanism but this has to be experimentally addressed (197). In conclusion, LCN2 has diverse functions in the cross-talk of different cell types in the TME and in cellular iron metabolism.

## Cellular iron homeostasis

While circulating hepcidin levels have a major impact on the iron content of FPN1-expressing cells, additional mechanisms exist to maintain cellular iron homeostasis by balancing iron uptake, release and storage. The relative contribution of mechanisms for iron uptake is slightly different between cell types, though. For many of these, including EPs, enterocytes, B and T cells, TFR1 is assumed to be the quantitatively most important iron acquisition protein. TFR1 enables receptor-mediated endocytosis of TBI from the circulating iron pool (42). The endosome containing holo-TF and TFR1 is acidified, which results in dissociation of the complex into its components, TFR1, TF, and iron. Thereafter, ferric iron is converted to its divalent form by a reductase, such as STEAP3 (for six-transmembrane epithelial antigen of the prostate-3) before it can be shifted to the cytoplasm via DMT1 (198). DMT1 is also present in the cell surface membrane, where it mediates the uptake of ferrous iron in cooperation with the reductase duodenal cytochrome B (DcytB). After entry into the cytoplasm, ferrous iron is stabilized by a chaperone, PCBP2 (for Poly(RC) binding protein-2), until further utilization (199, 200). In the cytoplasm, labile iron can be incorporated into FT for storage. FT is a multimer that consists of 24 FTH and FTL subunits, assembled in cell-type specific proportions. In EPs and macrophages, FTH is the predominant subunit, possibly because these cells are exposed to the fastest iron fluxes and FTH is the only subunit carrying the ferroxidase activity essential for iron storage (201). In order to make FT-stored iron metabolically available again, FT needs to be degraded by an autophagosomal mechanism that relies on NCOA4 (for nuclear receptor coactivator-4) as cargo receptor (202, 203). After re-entry into the cytoplasmatic labile iron pool (LIP), iron can be used for metabolic or synthetic purposes, such as incorporation into iron-containing enzymes. Excess iron can also be exported out of the cytoplasm by the activity of FPN1. As mentioned above, the expression of many iron-handling proteins, such as TFR1, DMT1, FT, and FPN1 is fine-tuned at the post-transcriptional and translational level via IRP/IRE interaction (204, 205). The IRP system is of different importance for various cell types, though. For instance, it is essential for IECs whereas proper macrophage function in the absence of infectious agents does not require IRP (206, 207). However, putative functions of IRPs in TAMs have not been experimentally tested to date.

In many macrophage populations including TAMs, the uptake of aged or damaged RBCs by scavenger receptors contributes to iron acquisition to a substantial extent (208, 209). In addition, free Hb and heme are cleared from the circulation in complex with the plasma proteins haptoglobin and hemopexin, respectively (210, 211). This uptake is enabled by receptor-mediated endocytosis via CD163 and CD91, protects from oxidative damage by free Hb and heme and supplies macrophages with iron.

## Ferroptosis, a unique form of iron-induced cell death

Ferroptosis is a form of regulated cell death *sui generis* that is elicited by iron-induced lipid peroxidation (3). The early events of ferroptosis are interconnected with ferritinophagy because NCOA4-facilitated FT degradation can provide free labile iron (212) (Figure 2). In the subsequent series of events culminating in iron-induced cell death, reduced glutathione peroxidase (GPX)-4 activity results in glutathione depletion and thus in increased susceptibility to oxidative stress. The tumor suppressor protein p53 induces ferroptosis via modulation of cysteine/glutamate metabolism with a direct impact on cellular oxidative stress, and p53 mutations, encountered in many types of cancer, result in the loss of p53-driven ferroptotic activity and tumor cell survival (213). It is thus not surprising that GPX4, ferroptosis and cancer are linked in several tumor entities including breast cancer, sarcomas and B cell lymphomas (214). In addition, the established anti-neoplastic drug sorafenib induces ferroptosis in hepatocellular carcinoma cells *in vitro* and novel compounds targeting GPX4 are effective in renal cell carcinoma and diffuse large B-cell lymphoma *in vivo* (215). Therefore, pharmacological modification of ferroptosis is a novel pathway for the treatment of both hematologic and solid malignancies.

## Iron regulates cell proliferation, metabolism, and signaling in cancer

As stated above, iron is essential for cell division and basic metabolic processes and hence indispensable for living cells including malignant ones. From a canonical point of view, cancer cells frequently demonstrate excessive proliferation rates and high metabolic turnover, and are thus believed to require more iron than their non-malignant counterparts. This notion is supported by concomitant visualization of iron content in tumor tissue and the physiological storage organs spleen and liver. For example, iron accumulation in mammary tumors of mice go hand in hand with depletion of systemic stores, suggesting active iron mobilization to satisfy cancer cell needs (216). Overexpression of FT, TFR1, and DMT1, dysregulation of IRPs and of the FPN1-hepcidin axis in favor of an iron-loading phenotype of cancer cells is linked to accelerated tumor progression (217–226). In line with this concept, application of iron chelators, dietetic iron depletion, and interference with the hepcidin-FPN1 dyad to withdraw iron from malignant cells is successful in cancer therapy *in vivo* and *in vitro* (227–233). Of note, phlebotomy has been demonstrated to reduce the prevalence of and death from cancer, too (234, 235). Other reports, however, contradict this point of view and demonstrate that increasing cellular and systemic iron stores may, paradoxically, keep tumor progression at bay (236–238). Hence, it is likely that an equilibrium of iron levels that meets metabolic needs but still does not cause cellular damage, impair oncogenic signaling or induce ferroptosis has to be established in cancer cells to sustain disease progression.

There are several mechanisms by which iron influences tumor cell growth in a positive or negative manner: (i) as a catalyst in non-enzymatic reactions of ROS generation, (ii) as a cofactor of enzymes involved in cell division like ribonucleotide-diphosphate reductase, (iii) as regulator of cell cycle control proteins, (iv) as a participant in pro- and anti-oncogenic signaling, and, finally (v), as a key component of the hypoxic response and metabolic as well as epigenetic re-programming, mediated by 2-oxoglutarate dioxygenases (239, 240). In a pre-malignant setting and in tumor progression, ROS generation and DNA damage resulting from it, may increase mutation rate and lead to a (more) malignant phenotype. This phenomenon is, indeed, reported in the animal model of *HFE*-associated hereditary hemochromatosis (HH), *Hfe*^−/−^ mice. There, high-iron diet increases the level of DNA damage in colon and mammary tissue and forms a possible mechanistic link between dietary iron and elevated colon carcinoma risk (241–244). On the other hand, IO and/or the occurrence of cellular labile iron in cancer cells may promote cell death via the same ROS-dependent mechanism, especially in combination with pro-apoptotic signaling and upon treatment with some chelating compounds (245, 246).

Stalling of the cell cycle and induction of apoptosis in iron-depleted cancer cells is a common observation made in multiple cancer cell lines (227, 231, 247, 248). The underlying mechanism is still not fully elucidated and can involve the distortion of cyclin expression and CDK (for cyclin-dependent kinase) activity pattern, as well as interference with the MDM2/p53 (for mouse double minute-2) pathway (231, 249–251). In addition, heme-iron directly interacts with p53 in normal hepatocytes and liver carcinoma cells and decreases its stability (252).

Iron has either attenuating or stimulatory effects on multiple signaling pathways. Iron affects NF-κB signaling by several mechanisms, such as increased peroxynitrite generation and subsequent tyrosine nitration in the inhibitory subunit of the NF-κB complex, IκBα, which may lead to its enhanced degradation (253). Iron on its own boosts IKK (for IκB kinase) activity as well (254). Of note, such enhancement of NF-κB, the master regulator of tumor-promoting inflammatory environment, may critically contribute to carcinogenesis (255). Iron plays a pivotal role in the stimulation of pro-inflammatory and oncogenic STAT3 signaling in colonic and hepatocellular carcinoma, too (256–258). And iron binding and activation of CDK1 with subsequent JAK2 (for Janus kinase-2) stimulation is a possible mechanistic explanation for this interplay. Iron is also shown to be involved in EMT, which is crucial for metastasis. This process, in turn, is under control of TGF-ß (for transforming growth factor-ß), WNT (for wingless-related integration site) and NOTCH signaling; all of which are stimulated by cellular iron loading and inhibited by its chelation (259–262).

The family of 2-oxoglutarate-dependent dioxygenases encompasses enzymes possessing ferrous ions in a non-heme and non-sulfur-cluster form in their active centers, requiring the citrate cycle intermediate 2-oxoglutarate as a cofactor for their activity and utilizing O_2_ as oxidizing substrate for catalyzed reactions. With those three components determining their activity, such proteins can be regarded as universal sensors of iron levels, energy status and oxygen levels in cells (263). Two most prominent subfamilies of 2-oxoglutarate-dependent dioxygenases are (i) Jumonji-type (JMJ) histone demethylases and (ii) HIF prolyl/aspartyl hydroxygenases. The link of the HIF hydroxygenases to iron metabolism of the normal and cancer cell will be discussed below in more detail.

JMJ histone demethylases are a group of enzymes sharing common structural motifs and a specificity for histone methyl-lysines (264, 265). As such, JMJ proteins are responsible for remodeling of the epigenetic landscape and tuning of the expression pattern in response to changes in energy status of the cell, oxygenation and iron levels (266–273). Importantly, a broad spectrum of the subfamily members displays overexpression and aberrant activity leading to oncogene expression, metabolic re-programming and cell cycle dysregulation in diverse malignancies (264, 274–279). In line, pre-clinical studies with murine models of prostate, mammary and lung cancer demonstrated high anti-neoplastic activity of JMJ-type demethylase inhibitors stressing the importance of JMJ-controlled epigenetic changes for malignancy (279–283). In addition, in mammary and ovarian cancer models, such blockers demonstrate interesting properties as they are particularly effective in targeting cancer stem cells, which often cannot be effectively reached by conventional chemo- and radiotherapy (282–284). Although ferrous iron constitutes a vital component of the JMJ-type enzymes, there are only few reports published to date, which systematically focus on the interplay between cellular iron status, JMJ enzyme activity and changes of epigenetic landscape of tumor cells. It is expected though that ID, e.g., due to chelation, should dampen activity of the JMJ proteins and, as a consequence, increase the general abundance of methyl-lysine repressing marks leading to downregulation of oncogene expression. There is indeed a major increase in histone methylation after iron chelation with deferroxamine (DFO), linking them to alterations in p21 and p53 expression (272). Interestingly, particular iron chelators specifically inhibit JMJ activity (285, 286); whole-genome epigenetic studies and reports on their anti-neoplastic properties are, however, still missing.

## Iron and the hypoxic response are linked in normal and malignant cells

Systemic and cellular adaptation to changeable concentrations of oxygen in the environment and within the tissue stays under control of evolutionary-conserved pathway of iron- and 2-oxoglutarate-dependent prolyl/aspartyl hydrogenases, PHDs, FIH (for factor inhibiting HIF), and HIFs (287–289). Under physiological O_2_ concentrations, both PHDs and FIH retain their full activity and hydroxylate proline residues in the oxygen-dependent domain (ODD) and asparagine residues in the transactivation domain (TAD) within HIF1α and HIF2α transcription factors. The PHD-mediated ODD hydroxylation causes ubiquitination by VHL (for von Hippel Lindau) Ub-ligase and targets the proteins for degradation. The TAD hydroxylation catalyzed by FIH, in turn, has no effect on protein levels but strongly inhibits its transcriptional activity. Under hypoxic conditions, activity of PHDs and FIH is diminished, enabling HIF1α and HIF2α accumulation, dimerization with the HIF1β coactivator, nuclear translocation and induction of the hypoxic response at the transcriptional level (289–292). Expression of HIF1α and HIF2α is tissue specific with substantial overlap in the repertoire of regulated genes. In tumor biology, HIF1α is believed to be of specific importance. The repertoire of HIF1α-induced genes encompasses angiogenic factors, such as VEGF and PDGF molecules, stroma and immune differentiation signals and a variety of amino acid and sugar transporters as well as enzymes of oxygen-independent energy metabolism (287, 289, 293–296). Activation of HIF1α at the early stages of carcinogenesis is crucial for the survival of the malignant cell since rapid cell division leads to shortages in oxygen and nutrient availability. HIF1α activation enables recruitment of blood vessels to the rudimentary neoplasm (so called angiogenic switch) and shifts the energy metabolism toward oxygen-independent glycolysis (so called glycolytic switch), with both these features being regarded as key properties of cancer (289, 295, 296).

Apart from oxygen concentration, iron availability is another factor determining activity of PHD and FIH hydroxylases, since redox-active iron ions are part of their catalytic centers. Along this line, modulation of cellular iron levels by chelation (e.g., with DFO) or iron supplementation can stimulate or inhibit the activity of these enzymes, respectively (263, 290, 297). Interestingly, the size of iron stores is inversely correlated with the degree of HIF-dependent respiratory response in humans as well (298). In DCs, FT has a pivotal role in the regulation of HIF1α as induction of FT by inflammatory stimuli decreases the LIP and hence iron available for PHD metalation and activity (299). As a result, scavenging of labile iron by FT causes HIF1α accumulation even at physiological O_2_ concentrations. Whether analogous mechanisms are active in other cell types, particularly malignant cells, remains to be investigated. Few reports, however, give some hints that the interplay of iron and HIF1α can bear significance for cancer biology. For impacting on cancer cell and tissue iron levels by means of TFR1 downregulation or dietary ID leads to increased HIF1α activity culminating in increased VEGF formation and cancer graft vascularization (10). In addition, dietary ID can accelerate mammary tumor growth and metastasis, and the activation of NOTCH signaling and HIF1α by ID is discussed as explanation of these paradoxical effects (237). In addition, low dietary iron intake affects tumor growth and susceptibility toward anti-VEGF therapy, and low iron diet results in a substantially better vascularization of the tumors (300). The net tumor growth is, however, decelerated following dietary ID as a result of slower tumor cell proliferation.

The question whether the functional iron/HIF1α interaction bears biological importance pertains to the management of AOC in the clinical setting as well. On one hand side, iron supplementation in any form (oral or intravenous) in cancer patients may bear a risk of faster progression due to stimulation of cancer cell proliferation as demonstrated in numerous pre-clinical reports (232, 259, 300). On the other, the same treatment may, in addition to improving quality of life of the patient, impair tumor metabolic adaptation and vascularization resulting in better outcome (10, 301). A novel class of drugs for the treatment of anemia, so called PHD inhibitors, which cause HIF1α stabilization, is discussed above (302, 303).

## Iron handling by tumor-associated macrophages and its implication for carcinogenesis

Tumors can be considered as organ-like structures with complex interactions between transformed and non-transformed cells. Stroma cells are needed to support the malignant potential of tumor cells, but tumors are additionally infiltrated by a wide range of immune cells, such as TAMs (304). TAMs can represent up to 50% of a tumor's mass and studies evaluated a significant link between TAM number and density with a poor prognosis of the underlying malignant disease (305–307).

Following the conventional M1/M2 classification, TAMs are typically M2-like cells and as such characterized by high expression of HMOX1, mannose receptor and scavenger receptor-A (308–313). M2-like TAMs preferentially home to hypoxic areas of the tumor, where their pro-tumoral activities are promoted (314). However, in cancer induced by chronic inflammation, TAMs can display an inflammatory M1-like phenotype or overlapping M1/M2 characteristics (315–317). In addition, specific microenvironmental signals may be important for different TAM activation states within the same tumor (294). In mammary tumors, two different microenvironments are infiltrated by different TAM subsets: (i) Sessile TAMs with high phagocytic capacity and expression of M2-like markers are present. (ii) Migratory TAMs are observed and produce EGF to attract cancer cells (318–321). The diversity and heterogeneity of TAMs in breast, lung, pancreas, brain and liver cancers is increasingly appreciated, too (322). It is thus likely that some TAM populations support tumor progression and development of metastasis, whereas others have anti-tumoral activities. Further insight in the microenvironmental stimuli, including iron, effector molecules and signaling pathways, that drive TAM heterogeneity within a tumor may be important to establish options to specifically target, inhibit or destroy pro-tumoral TAM populations in cancer patients.

As already stressed, macrophages residing in normal tissues can be regarded as “gate-keepers” of iron metabolism, i.e., cells which take up iron, store it in excess and export it to cover the needs of the surrounding cells. This role can also be supposed for TAMs in the TME and macrophage polarization dictates the way they are handling iron: whereas FT^high^, FPN1^low^ M1-like macrophages are predisposed to iron withdrawal, restriction and storage, the FT^low^, FPN1^high^ M2-like subtype promotes iron export and iron redistribution to the extracellular space. TAMs display a strongly M2-polarized phenotype in most types of malignancies and are ascribed such “iron-donating” features that may contribute to their tumor-promoting properties (323). In line with this model, human M2-skewed macrophages boost proliferation of human cancer cells *in vitro* in an iron- and FPN1-dependent manner (324). Data obtained with animal models and human tumor tissue further stress the “iron gate-keeper” and “iron donating” phenotype of TAMs. In murine primary lesions of the prostate or breast as well as in lung and brain metastasis, iron-storing macrophages can be visualized by magnetic resonance imaging (MRI) and histology (325, 326). In the human setting, iron deposition and expression of the iron turnover machinery (i.e., FPN1, hepcidin, TFR1, and FT) is significantly enriched in the macrophage and lymphocyte compartment (327, 328). Some indirect hints for the preferential active iron uptake by TAMs in the TME can be inferred from MRI, microscopy and cytometry studies using iron nanoparticles to specifically label this cell type (329–332). An interesting additional phenomenon is the fact that TAM-derived FT acts on malignant mammary epithelium as a growth factor (333). These growth-promoting effects of FT are, however, independent of its iron content.

The canonical view on iron homeostasis stresses the central role of the sole iron exporter FPN1 and its antagonist, hepcidin. In accord with it, FPN1, highly expressed on alternatively activated TAMs, should constitute the exclusive pipeline of the macrophage-tumor cell iron transfer. Its functionality may, however, not operate optimally under high systemic hepcidin levels in cancer patients and in the inflammatory, hepcidin-rich TME (219, 223, 327, 334). Importantly, increased hepcidin is unequivocally linked to accelerated tumor progression in experimental animals and in breast cancer individuals (219, 224, 225). This paradox suggests either a hepcidin-independent regulation of FPN1 in TAMs or the existence of alternative iron transport routes in the TME. The former possibility is supported by the data obtained in human breast carcinoma (327). There, elevated FPN1 levels in the macrophage infiltrate can be discerned in ductal carcinoma *in situ* (DCIS) and ductal carcinoma as compared to normal breast tissue. Concomitantly, the same stages of breast carcinogenesis demonstrate substantially increased hepcidin. The existence of FPN1-independent iron export can, in turn, be inferred from few reports identifying TAMs as the main source of LCN2 (335–337). Two reports dealing with the macrophage-epithelium iron transfer in mammary carcinoma describe a critical contribution of macrophage-secreted LCN2 to optimal proliferation and iron supply of cancer cells *in vitro* (338, 339) (Figure 2).

The great majority of literature on TAMs and iron refers to research on breast cancer. However, iron transfer in the TME may be subject to mechanisms specific to a given tumor entity. An interesting phenomenon, apparently contradicting the “iron-donating” phenotype of TAMs is observed in a murine lung carcinoma model. There, TAMs dwelling in hemorrhagic regions surprisingly demonstrate excessive iron loading and the classical M1 phenotype with a notable upregulation of NOS2 and toxicity against malignant cells (332). Mechanistically, such properties are provoked by ingestion of damaged RBCs reaching the tumor parenchyma via leaky tumor vasculature. Of great clinical interest, the M1 phenotype can also be induced by treatment of tumor-bearing mice with iron microparticles resulting in net tumor suppression (332).

Summarizing, TAMs, in parallel to “physiological” tissue-resident macrophages, can be regarded as the key nexus of iron homeostasis in the TME. These cells, however, can function both as tumor-promoting donors of the element and/or as iron-laden tumor cytotoxic leukocytes. For it remains open whether damaged RBCs, ingested apoptotic cells, which are widespread in the malignant milieu, TAM-derived siderophore-bound iron, FT or TBI from the systemic circulation pose such an iron source (332, 337).

## Iron controls T cell function

The microenvironment of solid tumors also contains tumor-infiltrating immune cells (TILs), including B cells and T cells, natural killer (NK) cells, neutrophils, myeloid-derived suppressor cells (MDSCs), and TAMs, and the function of all of these cells may be affected by iron (340–344).

For example, iron has immunosuppressive effects on T cell responses. According to early studies, the immune system and its circulating components are involved in the recognition and binding of metals as protection against metal toxicity, and the use of metals, such as iron, by bacteria or transformed cells (345). In line, in patients with thalassemia, iron influences the expansion of different T cell subsets (346). Furthermore, in patients with *HFE*-associated HH, a decrease in T cell numbers and activation defects can be observed, which may be causally linked to the toxic effects of free iron and oxidative stress (347). Additionally, abnormalities in the relative proportions of CD4^+^ and CD8^+^ subpopulations are described (348, 349). A similar impairment is observed in individuals receiving blood transfusions in the setting of TRIM and following intravenous iron infusions for the treatment of ID anemia or AOC as discussed above (350–353).

In general, there are different types of cancer in which iron has been implicated (354). However, very few reports address the influence of iron on cells of the adaptive immune system. The majority of studies deal with patients suffering from breast cancer. In these patients, a link between dysregulation of iron metabolism and progression of cancer exists (355). Experimental data indicate that a chronic failure in iron-dependent redox balance leads to the loss of tumor suppressors, oncogene expression and triggering of pro-oncogenic signaling, such as WNT and NF-κB pathways (259, 356–359). Several studies also point out that elevated iron stores are associated with increased risk for cancer development (360–362). However, many of these studies use serum FT as an indicator of iron loading which may be misleading because even in subclinical inflammation, FT levels may be increased by the action of cytokines, thus not accurately reflecting iron stores. Therefore, the association of high FT levels with the risk of cancer may in part reflect an inflammatory state as an expression of incipient cancer rather than a causative role of iron loading. Increased serum FT has been found to be associated with breast cancer risk, and FT levels in cancer-tissue are significantly increased in cancer specimens and correlate with higher degrees of tumor cell proliferation (363, 364). Functionally, FT secreted by TAMs is proposed to act as tumor growth factor and immunosuppressant (333, 365). However, the opposite functions of this protein on anti-tumor immunity are reported as well. The subcellular localization of FTH is important in triple negative breast cancer (366). This may be attributable to the fact that cytoplasmic FTH in tumor tissues regulates the MHC-I part of the antigen processing and presentation pathway and subsequently attracts CD8^+^ T cells to target tumor cells, whereas nuclear FTH supports the survival of cancer cells. Consequently, animal studies indicate that low iron nutrition and application of iron chelators moderate tumor growth and inhibit metastasis (367). Of note, FT can act as a pro-inflammatory mediator independent of iron availability, thereby affecting protein kinase C- and NF-κB-mediated signaling processes (368).

It is well-known that the proliferation of T cells requires iron, and intracellular iron stored in FT is thought to sustain proliferation of immune cells (369–371). In addition, a mutation in the gene encoding TFR1, *TFRC*, results in impaired T as well as B cell function (372). In line, ID reduces T cell numbers and impairs the activity of NK cells (373). Furthermore, iron chelation inhibits the production of IFN-γ, IL-2, and GM-CSF (for granulocyte-macrophage colony-stimulating factor) by T cells (374). On the other hand, patients suffering from IO secondary to ß-thalassemia have decreased CD4^+^ and increased CD8^+^ T cell numbers, while patients with *HFE*-associated HH show a trend to lower CD8^+^ T cells dependent on their HLA haplotype (347, 375– 377). Along with this, genetic deletion of FTH reduces the number of mature B cells and peripheral T cells in all lymphoid organs as a result of increased LIP and enhanced ROS formation (378). Therefore, a balanced iron metabolism is central for proper T cell function, but to which extent dysbalances affect the clinical course of cancer awaits further investigation.

## Putative roles of iron in cancer biology beyond cancer and immune cells

Iron may influence cancer biology independent of its effects on cancer and immune cells, for example by altering the microbiome or the function of stromal cells in the TME. The role of the microbiome in cancer development is increasingly appreciated (379). This is especially true of the pathogenesis of colon cancer, which is thought to be strongly affected by the intraluminal microflora of the gastrointestinal tract (380). Of note, the composition and the iron content of the diet influences the diversity of the gastrointestinal microbiome, which may have secondary effects on IECs and the mucosal immune system. (381–385). These alterations may impair GI barrier function, undermine colonization resistance and increase the risk of colon cancer, but probiotic supplementation may prevent these adverse effects (386). In addition, heme has pro-oxidative properties and acts as DAMP which is recognized by TLR4 (for toll-like receptor-4) and cryopyrin (387, 388). As a consequence, pro-inflammatory signaling is initiated in IECs and ECs, whereas myeloid cell functions tend to be impaired by heme excess (389–392).

Of interest, the microbiome also increases the potential of heme to cause lipid peroxidation, whereas depletion of bacterial commensals with broad-spectrum antibiotics reverses this effect (393). In addition, antibiotic treatment suppresses bacteria that impair the intestinal mucus barrier thereby preventing heme's proliferation-inducing effect on IECs (394). Moroever, heme causes mutations in genes promoting colon cancer, such as APC (for adenomatous polyposis coli) and KRAS (395). These data suggest that an interaction between diet, the microbiome and the intestinal epithelium determines the susceptibility to colon cancer, indeed.

Various species of enterobacteriaceae produce siderophores in the GI lumen. Siderophores scavenge iron, which results in iron depletion of IECs and subsequent HIF1α activation (396). Apparently, activation of HIF1α promotes GI inflammation and alters WNT signaling but does not directly contribute to the pathogenesis of colon cancer (397–399). In contrast, HIF2α activation in IECs stimulates their proliferation rate and promotes neutrophil recruitment to the TME, thereby facilitating the occurrence of colon carcinoma (400, 401). This is also relevant for systemic iron homeostasis because HIF2α controls DMT1 and DcytB expression and induces FPN1 expression in response to ID (402–404). In addition, activation of HIFs results in VEGF production, which acts on ECs and is a prerequisite for tumor angiogenesis. Thus, the HIF-VEGF axis is another potential pathway linking intestinal dysbiosis to pro-oncogenic behavior in neoplastic and stromal cells.

In addition, LCN2 affects the balance between bacteria that do or do not utilize LCN2-susceptible siderophores, such as catecholate-type ones (405). In the absence of LCN2, *Alistipes* species outcompete other commensals because of their ability to secrete enterobactin, resulting in dysbiosis that promotes colorectal carcinogenesis (406).

In conclusion, dietary iron affects IECs, mucosal immune cells and the microbiome and modulates their interaction, thus promoting colorectal carcinogenesis.

## Discussion

Our tools to manipulate systemic iron homeostasis have been evolving over the last couple of years. Medications for the treatment of ACD have direct or indirect effects on iron homeostasis and include AHS, calcium channel blockers, cytokine antagonists, PHD inhibitors, kinase inhibitors, ESAs and multiple iron preparations (302, 407–411). However, not all of these compounds are well-studied in cancer patients yet. Also, the local effects that these medications may have in the TME and therefore, in the medium-term, on the underlying malignant disease are largely unknown. We thus need to gain further insight into the effects of such treatments on the composition of the TME, the immune control of cancer, the metabolic re-programming of immune and cancer cells, their impact on cellular stress and proliferative/apoptotic/ferroptotic responses, along with off-target effects of such treatments linked to e.g., tumor vascularization and development of distant metastasis. We are also in a need to gather further knowledge on the effect of AOC correction by any treatment on the subsequent course of the malignant disease along with a personalized view depending on the tumor entities and specific factors of the individual patient.

It is only in recent years that we have begun to unscramble the complex, reciprocal and countless interconnections between iron metabolism and cancer as reviewed herein. Observations made in *in vitro* systems, co-culture models, organoids, and small animal models need to be carefully translated to the human setting. Novel technology, such as laser dissection microscopy, multi-laser flow cytometry and single-cell RNA sequencing on human cancer tissue may help in this translation process. Eventually, we face the challenge to close existing knowledge gaps and connect the dots to see the complete picture of the many roles of iron in cancer occurrence, progression and treatment for the sake of improved care for hemato-oncologic patients.

## Author contributions

CP-O wrote and edited the manuscript. PT wrote and edited the manuscript. VP discussed and edited the manuscript. GW provided the concept and wrote the manuscript. MN provided the concept, wrote the manuscript and drew the figures.

### Conflict of interest statement

The authors declare that the research was conducted in the absence of any commercial or financial relationships that could be construed as a potential conflict of interest.

## Abbreviations

ACD: anemia of chronic disease
AHS: anti-hepcidin strategy
AOC: anemia of cancer
APC: adenomatous polyposis coli
ATP: adenosine triphosphate
BMP6: bone morphogenic protein-6
CD: cluster of differentiation
CHOP: CCAAT-enhancer-binding protein homologous protein
CDK: cyclin-dependent kinase
CKD: chronic kidney disease
CML: chronic myeloid leukemia
CREB-H: cyclic AMP response element-binding protein H
DAMP: danger-associated molecular pattern
DC: dendritic cell
DCIS: ductal carcinoma *in situ*
DFO: deferroxamine
DMT1: divalent metal transporter-1 AKA SLC11A2
DNA: deoxyribonucleic acid
EC: endothelial cell
EDTA: ethylenediaminetetraacetic acid
EGF: epidermal growth factor
EMT: epithelial-mesenchymal transition
EP: erythroid progenitor
EPO: erythropoietin
ER: endoplasmatic reticulum
ERFE: erythroferrone
ERK: extracellular signal-regulated kinases
ESA: erythropoiesis-stimulating agent
FIH: factor inhibiting HIF
FPN1: ferroportin-1 AKA SLC40A1
FT: ferritin
FTL: ferritin light chain
FTH: ferritin heavy chain
GAPDH: glyceraldehyde-3-phosphate dehydrogenase
GDF15: growth differentiation factor-15
GM-CSF: granulocyte-macrophage colony-stimulating factor
GPX4: glutathione peroxidase-4
Hb: hemoglobin
HFE: hemochromatosis-associated gene/protein
HJV: hemojuvelin
HH: hereditary hemochromatosis
HMOX1: heme oxygenase-1
ID: iron deficiency
IEC: intestinal epithelial cell
IFN-γ: interferon gamma
IKK: IκB kinase
IL: interleukin
IO: iron overload
IRE: iron-responsive element
IRP: iron-regulatory protein
JAK: Janus kinase
JMJ: Jumonji-type protein
LCN2: lipocalin-2 AKA NGAL (for Neutrophil gelatinase-associated lipocalin)
LCNR: lipocalin-2 receptor AKA SLC22A17
LF: lactoferrin
LIP: labile iron pool
LPS: lipopolysaccharide
MAP kinase: mitogen-activated protein kinase
MDM2: mouse double minute-2
MDS: myelodysplastic syndrome
MDSC: myeloid-derived suppressor cell
MM: multiple myeloma
MRI: magnetic resonance imaging
mRNA: messenger ribonucleic acid
miRNA: micro ribonucleic acid
NCOA4: nuclear receptor coactivator-4
NF-κB: nuclear factor kappa-light-chain-enhancer of activated B cells
NOS2: nitric oxide synthase-2 AKA inducible NOS
NSCLC: non-small cell lung cancer
NK: natural killer
NKT cell: natural killer T cell
NTBI: non-transferrin-bound iron
ODD: oxygen-dependent domain
PAMP: pathogen-associated molecular pattern
PCBP2: poly(RC) binding protein-2
PDGF: platelet-derived growth factor
PHD: prolyl HIF dioxygenases
RBC: red blood cell
RCT: randomized controlled trial
RNS: reactive nitrogen species
ROS: reactive oxygen species
SCARA5: scavenger receptor class A member-5
SLC: solute carrier
SMAD: homologs of Sma and Mad (mothers against decapentaplegic) proteins
STAT: signal transducer and activator of transcription
STEAP3: six-transmembrane early antigen of the prostate-3
TAD: transactivation domain
TAM: tumor-associated macrophage
TBI: transferrin-bound iron
T_C_ cell: cytotoxic T cell
TFR: transferrin receptor
TGF-ß: transforming growth factor-ß
T_H_ cell: T helper cell
TIL: tumor-infiltrating lymphocyte
TIM2: T cell immunoglobulin and mucin domain protein-2
TLR4: toll-like receptor-4
T_REG_ cell: regulatory T cell
TME: tumor microenvironment
TNF: tumor necrosis factor
TRIM: transfusion-related immune modulation
VEGF: vascular endothelial growth factor
VHL: von Hippel-Lindau tumor suppressor
WNT: wingless-related integration site.
